# Racial discrimination and disease damage among African American women with systemic lupus erythematosus

**DOI:** 10.1186/ar4629

**Published:** 2014-09-18

**Authors:** David H Chae, Christina M Drenkard, Tené T Lewis, S Sam Lim

**Affiliations:** 1 School of Public Health, University of Maryland at College Park, College Park, MD, USA; 2 School of Medicine, Emory University, Atlanta, GA, USA; 3 School of Public Health, Emory University, Atlanta, GA, USA

## Background

African American women with SLE experience faster progression and worse consequences of disease compared with their White counterparts. This study sought to examine whether self-reported routine experiences of discrimination, as a source of psychosocial stress, is associated with disease damage among African American women with SLE.

## Methods

Participants were 578 African American women in the Georgians Organized Against Lupus study, a population-based cohort of SLE patients in Atlanta, GA, USA. Disease damage was assessed using the Self-Administered Brief Index of Lupus Damage (SA-BILD), a validated, patient-reported measure of organ damage since the onset of SLE. Discrimination was assessed using the Everyday Discrimination Scale, a widely used measure of routine experiences of unfair treatment. Ordinary least-squares regression analyses were used to examine the outcome of SA-BILD score by the primary predictors: unfair treatment, racial discrimination attribution, and their interaction, controlling for age and years since SLE diagnosis.

## Results

**Figure 1:**
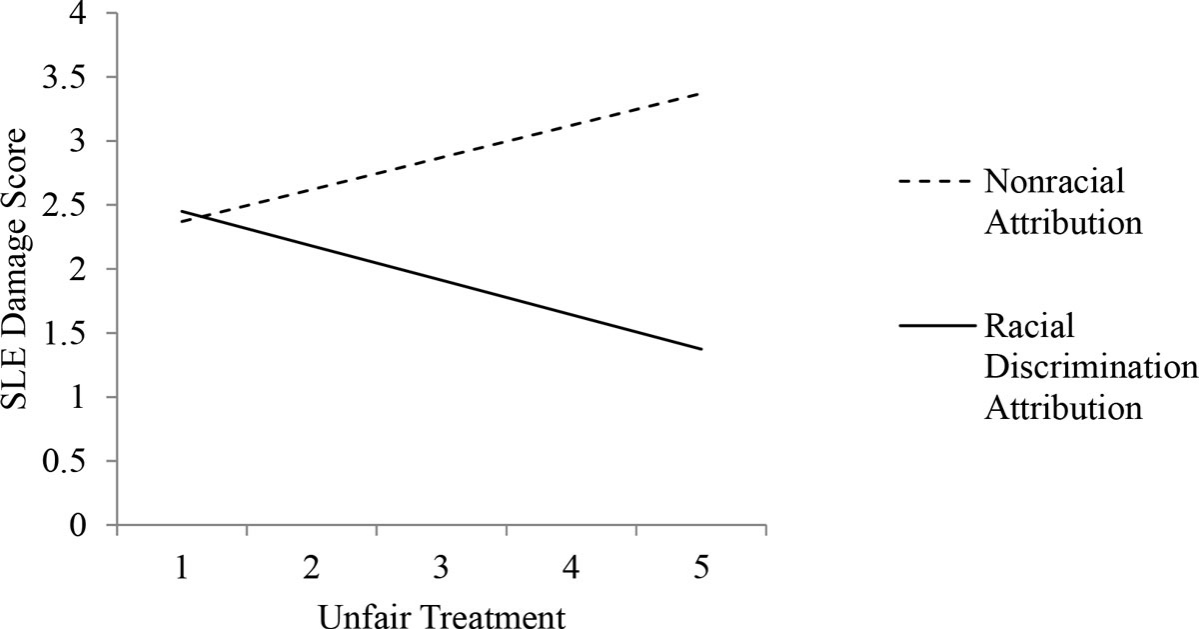
**Predicted disease damage score by attribution to racial discrimination among African American women with systemic lupus erythematosus (SLE) reporting any unfair treatment in the Georgians Organized Against Lupus (GOAL) study (*n *= 578; 2011 to 2012)**.

The average SLE damage score in our sample was 2.3 (SD = 2.4), and the mean years since initial diagnosis was 13.6 years (SD = 9.3). The mean unfair treatment score was 1.92 (SD = 0.95), indicating that on average participants reported experiencing each of the forms of unfair treatment approximately once a year. A total of 159 participants (27.6%) reported not experiencing any unfair treatment. Among participants reporting any unfair treatment, most did not make an attribution of racial discrimination (*n *= 258 compared with *n *= 146). Age (*r *= 0.23, *P *< 0.001) and years since diagnosis (*r *= 0.25, *P *< 0.001) were significantly correlated with SLE damage. Reports of unfair treatment and making an attribution to racial discrimination were not significantly associated with SLE damage. In multivariable regression analyses controlling for age and years since diagnosis, we found a significant interaction between unfair treatment and attributions to racial discrimination (*b *= -0.52, SE = 0.24, *P *= 0.03). Greater unfair treatment attributed to nonracial causes was associated with higher SA-BILD score, whereas unfair treatment attributed to race showed an inverse association (Figure 1).

## Conclusions

This study highlights the role that social stressors have in contributing to the progression of SLE and is the first to examine whether unfair treatment and racial discrimination are associated with disease damage among African American women with SLE. Consistent with findings from studies on discrimination and other health outcomes, these results suggest more complex, interactive rather than direct associations with SLE damage, with differential relationships being found between those who attributed unfair treatment primarily to racial discrimination versus those who did not.

